# A Cross-Sectional Study of the Knowledge, Practice, and Attitude Towards Skin-Lightening Products Among the General Population in the Western Region of Saudi Arabia

**DOI:** 10.7759/cureus.34069

**Published:** 2023-01-22

**Authors:** Shahad Bamerdah, Omar S Alhothali, Bushra M Aldajani, Logain Alghanemi, Nouf T Mleeh

**Affiliations:** 1 Faculty of Medicine, Umm Al-Qura University, Makkah, SAU; 2 Medicine and Surgery, Umm Al-Qura University, Makkah, SAU; 3 Dermatology, King Abdulaziz University Hospital, Jeddah, SAU

**Keywords:** saudi arabia, knowledge, practice, general population, skin lightening

## Abstract

Introduction

Skin bleaching is a growing phenomenon worldwide and is becoming an increasing problem. Several skin-lightening products (SLPs) containing mercury, hydroquinone, and corticosteroids have impacted serious dermatological, nephrological, and neurological side effects. There is relatively little regulation, and the products are easily accessible and inexpensive. Justifications and beliefs for the use of these products vary from culture to culture, and there is little previous research on the use and abuse of skin-lightening cosmetics among Saudi women. This study examines the knowledge, attitudes, and practices of the public in the western region of Saudi Arabia regarding SLPs to understand the situation better.

Methodology

An observational, cross-sectional, questionnaire-based study was conducted over two months between July and August 2022. A 29-question survey was used to collect data from the general population. The study included all women residing in the western region of Saudi Arabia. Non-Arabic speakers were excluded. RStudio (R version 4.1.1) was used to analyze the data.

Results

A total of 409 participants were included in this study; In general, 146 (35.7%) of the participants said they had ever used an SLP. More than two-thirds (67.1%) had been using them for less than a year. In terms of the most common site of SLPs application, women reported applying the products to the skin of their face (74.7%), elbows (47.3%), and knees (46.6%). Use of SLPs differed significantly across participants' ages, with the proportion of SLP users in the 20-30 age category significantly higher than non-users (50.7% vs. 36.9%, p=0.017), and non-users were more common than users within the age category >50 years. In addition, the proportion of SLP users relative to educational level was significantly higher among participants with a bachelor's degree than the proportion of non-users (69.2% vs. 54.0%, p = 0.009).

Conclusions

The results of this research show that Saudi women frequently utilize topical lightening products. Therefore, regulation and controlling the use of bleaching products is essential, as is educating women about the risks involved with this practice. The misuse of bleaching products should decline with greater awareness.

## Introduction

Skin bleaching is referred to as skin whitening, skin fairness, skin lightening, skin depigmentation, skin toning, and skin lighting. It is a cosmetic method that includes the use of harmful substances (e.g., mercurials) or using skin-whitening chemicals (such as topically applied steroids) to alter an individual's biological skin tone [1-3]. Several research papers have looked into the female's reasons for being involved in skin bleaching. Those studies concluded that the most common reasons are to become light-skinned, pretty, and more European in appearance. Other motivations include getting rid of pustules, redness, and skin problems, and impressing peers and males [4].

Skin bleaching is a growing worldwide phenomenon and is becoming a more significant issue. According to the World Health Organization, dangerous skin bleaching without professional counseling has become a public health crisis [5]. Several skin-lightening products (SLPs) contain mercury, hydroquinone, and corticosteroids and have influenced serious dermatological, nephrological, and neurological side effects [6]. More than 60% of those using SLPs may suffer from at least one complication [7].

Possible effects of hydroquinone use for skin lightening include post-inflammatory dyspigmentation, irritant dermatitis, as well as allergic contact dermatitis [7]. One significant concern addressed in several of these papers is hydroquinone-induced ochronosis. This side effect is most common in Africa, where skin whitening is a common cultural practice. Among the 789 cases of reported exogenous ochronosis in the literature, 756 were from Africa [8,9].

The prolonged consumption of corticosteroids (more than three weeks), particularly on thin skin like the face and flexural areas, has been linked to several side effects, including skin atrophy, telangiectasias, allergic contact dermatitis, striae, acne or rosacea-like eruption, poor wound healing, easy bruising, and hypertrichosis. In addition, ocular disorders (cataracts, glaucoma, eye infections, and blindness) have been linked to the use of topical corticosteroids on the face, particularly the lids, as well as aseptic osteonecrosis [10,11].

The importation and marketing of skin-whitening substances have been restricted or heavily regulated in many African, Asian, European, and North American countries due to the linked effects [12], where it is only available by prescription. However, it is still a cosmetic product in other countries like Saudi Arabia, even in concentrations higher than acceptable (2%) [6].

Skin whitening has been reported in many countries; half of the population in Korea, Malaysia, and the Philippines use some skin-lightening treatment, 77% of Nigerian women use SLPs regularly, and 61% of the skincare market in India consists of SLPs [5,13]. In addition, skin whitening is becoming more prevalent in Saudi Arabia, with nearly 40% of women actively using skin-whitening products [14].

A study of Saudi women conducted in the Riyadh region assessed their knowledge and practice of skin lightening and discovered that 45.4% of the women did not believe that using lightening creams could harm their skin, and 80% did not agree that using lightening products could harm a user's overall health [15]. Another Saudi study was carried out in Al-Madinah Al-Munawwarah, and it was discovered that the most common side effects of discontinuation were the restoration of normal skin color (44.3%), and darker skin (27%), skin dryness (20%), and rash (9.6%). Only 30.2% of their participants believe mercury harms their health [16]. These findings are concerning and present a significant challenge to overcome.

According to our database search, only a few researches in this regard were done in different regions in Saudi Arabia [6,15-17]. However, no such study was conducted in the western region of Saudi Arabia; therefore, we planned to conduct this study to assess the knowledge, practice, and attitudes toward SLPs among the general population in the western region of Saudi Arabia.

## Materials and methods

This observational, cross-sectional analytical study was conducted using a self-administered questionnaire. The study targeted the general population of the western region of Saudi Arabia and spanned two months between July and August 2022. We included adult females aged 18 and above. Individuals who didn't speak Arabic were excluded from the study. Participants were selected using a non-sampling convenience sampling technique. Based on a review of similar studies, the research team developed a 29-item questionnaire divided into four sections to accomplish the research objectives. The first section (four questions) focused on sociodemographic characteristics (age group, educational level, marital status, skin tone). The second section (four questions) covered perceptions toward a lighter skin tone. The third section (16 questions) is about the prevalence of the use of SLPs and knowledge of SLPs, reasons for use, application practice, and product sources. The fourth section (five questions) assessed the usage of a triple mixture. The data were collected through an online Google survey distributed through social media platforms. Participants completed the survey anonymously and voluntarily. The questionnaire was designed in English and translated into Arabic to fit the country's native language. We used OpenEpi (Version 3.0, www.OpenEpi.com) for sample size calculation: a minimum sample size of 385 was required for the study, considering a 95% confidence interval (CI) and anticipated frequency of 50%, and design effects of 1.

Statistical analysis

In the current study, statistical analysis was carried out using RStudio (R version 4.1.1). We used frequencies and percentages to express the categorical data, and numerical data were presented as the median and interquartile range (IQR). A multiple-response analysis was used to analyze items with more than one available selection. A one-sample proportions test with continuity correction was used to express the prevalence of using SLPs and the respective 95% CI. Factors associated with SLPs were assessed using Pearson's Chi-squared test or Fisher's exact test, whenever applicable. The significantly associated variables from the association analysis were entered in a binary logistic regression model to assess the predictors of using SLPs, and the results were presented as odds ratios (ORs) and 95%CIs. A p-value indicated statistical significance.

Ethical considerations

The Medical Research Ethics Committee approved this study at Umm Al-Qura University (approval number: HAPO-02-k-012-2022-06-1123). Consent to participate was taken from participants electronically.

## Results

Initially, we received responses from 559 participants. One participant declined to participate, and the responses of 29 males were received on the online platform. These responses were excluded before the analysis. Additionally, we excluded the records of 120 participants with missing responses to the primary outcome variable (use of SLPs). Eventually, the records of 409 participants were analyzed.

Sociodemographic characteristics and their association with using SLPs

Less than half of the participants were aged 20 to 30 years (41.8%), and all of them were residing in the western region. More than half of the respondents were single (60.6%) and had obtained a bachelor's degree (59.4%). The most common skin tones were light brown (46.2%) and light (39.4%, Table 1).

In general, 146 participants indicated that they had ever used an SLP, representing 35.7% (95%CI, 31.1% to 40.6%) of the overall sample. The use of SLPs differed significantly based on participants' age, where the proportion of SLP users was significantly higher than non-users within the 20-to-30-year age category (50.7% vs. 36.9%, p = 0.017) and non-users were more frequent than users within the >50-year-age category. Furthermore, based on the educational level, the proportion of SLP users was significantly higher than non-users among the participants who had obtained a bachelor's degree (69.2% vs. 54.0%, p = 0.009). No significant differences in SLP use were noted based on marital status and skin tone (Table 1).

**Table 1:** Sociodemographic characteristics and their association with using skin-lightening products. SLPs: skin-lightening products

Parameter	Category	Overall, N = 409	Use of SLP		
			No, N = 263	Yes, N = 146	p
Age	< 20	84 (20.5%)	61 (23.2%)	23 (15.8%)	0.017
	20 to 30	171 (41.8%)	97 (36.9%)	74 (50.7%)	
	31 to 40	79 (19.3%)	50 (19.0%)	29 (19.9%)	
	41 to 50	44 (10.8%)	29 (11.0%)	15 (10.3%)	
	> 50	31 (7.6%)	26 (9.9%)	5 (3.4%)	
Residence Area	Western region	409 (100.0%)	263 (100.0%)	146 (100.0%)	>0.999
Marital Status	Single	248 (60.6%)	165 (62.7%)	83 (56.8%)	0.619
	Married	138 (33.7%)	85 (32.3%)	53 (36.3%)	
	Divorced	18 (4.4%)	10 (3.8%)	8 (5.5%)	
	Widow	5 (1.2%)	3 (1.1%)	2 (1.4%)	
Level of Education	Illiterate	4 (1.0%)	3 (1.1%)	1 (0.7%)	0.009
	Below high school	20 (4.9%)	18 (6.8%)	2 (1.4%)	
	High school or diploma	129 (31.5%)	92 (35.0%)	37 (25.3%)	
	Bachelor degree	243 (59.4%)	142 (54.0%)	101 (69.2%)	
	Post-graduate degree	13 (3.2%)	8 (3.0%)	5 (3.4%)	
Skin Tone	Light	161 (39.4%)	108 (41.1%)	53 (36.3%)	0.610
	Light brown	189 (46.2%)	116 (44.1%)	73 (50.0%)	
	Dark brown	39 (9.5%)	27 (10.3%)	12 (8.2%)	
	Black	20 (4.9%)	12 (4.6%)	8 (5.5%)	

We incorporated age and educational levels as independent variables in a multivariate logistic regression model to further reveal the independent predictors of SLP use. Results showed that participants aged >50 were less likely to use SLPs (OR = 0.30, 95%CI, 0.09 to 0.91, p = 0.045, Table 2).

**Table 2:** Results of the regression analysis to assess the predictors of using skin-lightening products. CI: confidence interval; OR: odds ratio; Ref: reference category

Parameter	Category	OR	95% CI	p
Age	< 20	Ref	Ref	
	20 to 30	1.19	0.61, 2.34	0.615
	31 to 40	0.94	0.45, 1.98	0.878
	41 to 50	0.8	0.33, 1.90	0.622
	> 50	0.3	0.09, 0.91	0.045
Level of Education	Illiterate	Ref	Ref	
	Below high school	0.14	0.01, 4.11	0.187
	High school or diploma	0.53	0.05, 11.9	0.612
	Bachelor degree	0.92	0.09, 20.4	0.949
	Post-graduate degree	0.96	0.08, 24.2	0.978

Participants' perceptions towards lighter skin tone

**Figure 1:**
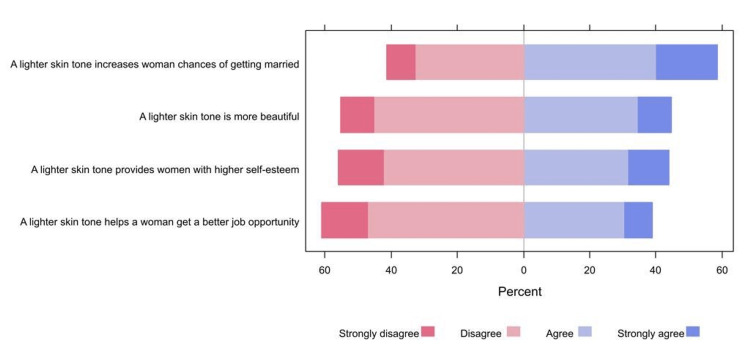
The percentages of participants’ responses regarding their perceptions towards a lighter skin tone (n=409).

More than half of the respondents (58.7%) agreed or strongly agreed that a lighter skin tone increases a woman's chances of getting married, whereas 44.7% agreed or strongly agreed that a lighter skin tone is more beautiful. On the other hand, 61.1% of the respondents disagreed or strongly disagreed that a lighter skin tone helps a woman get a better job opportunity (Figure 1).

Patterns of using SLPs

**Figure 2:**
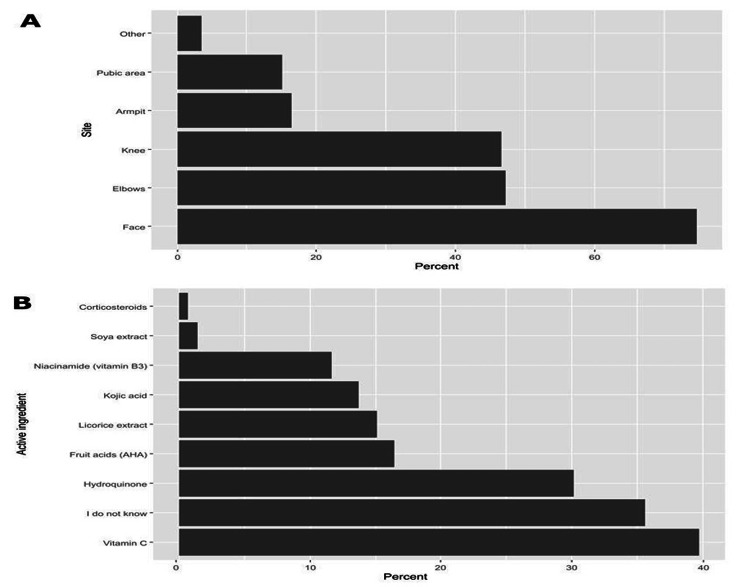
The percentages of participants’ responses regarding the body region on which SLPs are applied (A) and the active ingredient(s) in the used SLPs (B). The percentages were based on a total of 146 participants who confirmed the use of SLPs. SLPs: skin-lightening products

More than two-thirds out of 146 (total number of participants who ever used SLPs) have been using these products for less than a year (67.1%) and used to buy these products from the pharmacy (72.6%). Additionally, more than half of them were using the products in the evening (63.7%), and these products were primarily used for hyperpigmentary disorders (such as melasma) among 58.9% of the respondents. Notably, physicians' advice was the most common source of using SLPs (49.3%), followed by advice from pharmacists (47.9%) and friends (36.3%, Table 3). Regarding the most common site of SLPs application, women stated that they applied the products on the skin of the face (74.7%), elbows (47.3%), and knees (46.6%, Figure 2A). The applied skin products were mainly composed of vitamin C (39.7%) and hydroquinone (30.1%), while 35.6% of the participants did not know the active ingredients of SLPs (Figure 2B).

**Table 3:** Patterns of using skin-lightening products among the participants who indicated the use of products (n=146). SAR: Saudi Riyals; SLPs: skin-lightening products *An asterisk indicates a multiple-response item

Parameter	Category	N (%)
The period since SLPs have been used	Less than a year	98 (67.1%)
	More than a year	48 (32.9%)
The time at which SLPs are applied	Morning	3 (2.1%)
	Evening	93 (63.7%)
	Morning and evening	40 (27.4%)
	Other times	10 (6.8%)
Where products were bought	Pharmacy	106 (72.6%)
	Small beauty stores	30 (20.5%)
	All of the above	8 (5.5%)
	Other	2 (1.4%)
Amount spent on SLPs each month (SAR)	< 50	22 (15.1%)
	50 to 110	51 (34.9%)
	110 to 150	31 (21.2%)
	> 150	42 (28.8%)
The reason for which SLPs are used	The face has a hyper pigmentary disorder like melasma	86 (58.9%)
	Having a dark skin tone and the preference of a lighter skin tone	5 (3.4%)
	Both reasons	38 (26.0%)
	Other	17 (11.6%)
The choice of SLPs is based on*	Advice from a physician	72 (49.3%)
	Advice from a pharmacist	70 (47.9%)
	Advice from a friend	53 (36.3%)
	TV ads	17 (11.6%)
	Advice from the seller in small beauty stores	16 (11.0%)
	Other	18 (12.3%)

Women's attitudes towards SLPs

The majority of SLP users (n=146) agreed or strongly agreed that sunscreen should be applied while going outside, particularly during the use of SLP (94.6%), and that the active ingredient is a crucial element for the selection of SLPs (90.4%). On the other hand, approximately one-quarter of the participants disagreed or strongly disagreed that the product price is an essential criterion while selecting an SLP (28.8%) and that the used SLP gave the anticipated results (24.6%, Table 4).

**Table 4:** Women’s attitudes towards skin-lightening products.

Parameter	Strongly disagree	Disagree	Agree	Strongly agree
The price is an important criterion for selecting the skin-lightening product you use	1 (0.7%)	41 (28.1%)	71 (48.6%)	33 (22.6%)
The active ingredient is an important criterion for selecting the skin-lightening product you use	2 (1.4%)	12 (8.2%)	80 (54.8%)	52 (35.6%)
You have to apply sunscreen when using skin-lightening products if you went outside during the day	2 (1.4%)	6 (4.1%)	62 (42.5%)	76 (52.1%)
The effect of sun exposure counteracts the effect of skin-lightening products	3 (2.1%)	12 (8.2%)	63 (43.2%)	68 (46.6%)
Skin-lightening products may cause undesirable side effects for the skin	4 (2.7%)	31 (21.2%)	79 (54.1%)	32 (21.9%)
The skin-lightening product you are using or have used gave you the results you wanted	4 (2.7%)	32 (21.9%)	89 (61.0%)	21 (14.4%)

Women's practice of using the triple mixtures and hydroquinone-containing SLPs

**Figure 3:**
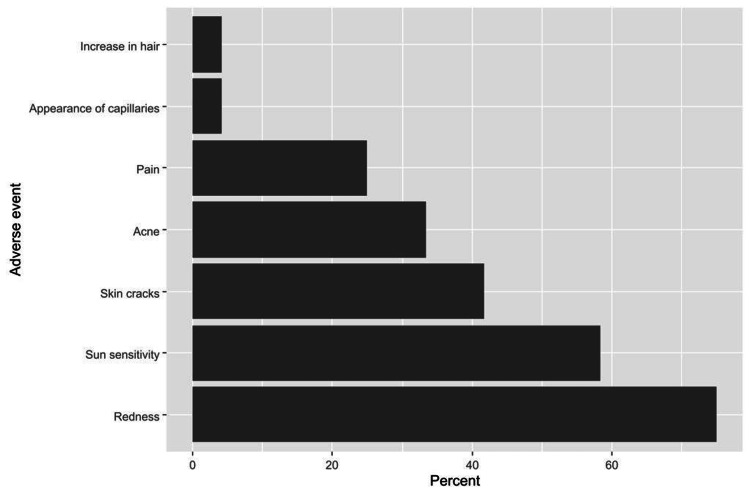
The percentages of adverse effects caused by triple mixtures among the participants who reported at least one adverse effect (n=24).

Only 13.7% of women who used SLPs corroborated that it is possible to apply a bleaching product containing hydroquinone for >3 months, while a considerable proportion of them did not know about that possibility (65.1%). Regarding the use of triple mixtures for skin lightening (which contain Hi Quin cream, Acretin cream, and Alfacort cream), 30.8% of women indicated using these mixtures. Of them, 55.6% used the mixture without physicians' consultation, and 75.6% knew the types of active ingredients in the mixtures (Table 5). Importantly, out of the women who had used the triple mixtures (n=45), 24 participants experienced at least one adverse event (53.3%). The most common adverse events included redness (75.0%), sun sensitivity (58.3%), and skin cracks (41.7%, Figure 3). Interestingly, half of the women with an adverse event ignored the complication (50.0%), whereas 20.8% visited a physician, 16.7% went to a pharmacy, and 12.5% talked to a friend/relative to discuss how to deal with the complication.

**Table 5:** Women’s practice of using the triple mixtures and hydroquinone-containing SLPs. *The records are based on the responses of 45 participants who had ever used the triple mixture; otherwise, the responses are based on the responses of 146. SLPs: skin-lightening products

Parameter	Category	N (%)
It is possible to use a bleaching product containing hydroquinone for more than three months	No	31 (21.2%)
	Yes	20 (13.7%)
	Do not know	95 (65.1%)
Using the triple mixture	Yes	45 (30.8%)
Using the mixture without consulting a doctor*	Yes	25 (55.6%)
Know the types of active substances in the triple mixture*	Yes	34 (75.6%)

## Discussion

Given the limited information on skin-lightening use in our region and our knowledge of the indexed literature, this examines skin-lightening practices and perceptions in the western region of Saudi Arabia. This study found that about a third (35.7%) of Saudi women engage in skin-lightening practices. Therefore, this study's finding underscores the importance of investing in primary prevention to reduce a potential long-term psychological, medical, and economic burden on population health and healthcare.

In the current study, there were significant differences in the number of people using SLPs depending on the age of the participants, while in the 20-30 age group, the percentage of SLPs users was significantly greater than non-users (50, 7% vs. 36.9%, p = 0.017). In the over-50 age group, non-users were more common than users. This finding is consistent with a study conducted in Ghana, which showed that participants under the age of 40 were 1.42 times more likely to use skin-lightening cosmetics than those over that age [18]. However, these results do not support previous research reported in the northern region of Saudi Arabia, where the study showed no significant correlation between those who use SLPs and those who don't, according to their age group [6].

In addition, the proportion of SLP users among bachelor graduates was significantly higher than among non-users in relation to educational level (69.2% vs. 54.0%, p = 0.009). Consistent with the present results, previous studies have shown a significant difference in education level; out of a total of 375 college-educated participants, 65.7% were skin whitening users, comparable to a total of 230 students using 34.3% skin whitening users [6].

In our study, among those who reported using SLPs (n=146), more than two-thirds (67.1%) had been using them for less than a year. In a study conducted in South Africa, the majority (62%) used a lightening cream for many years. A quarter of the women said they had used a lightning cream for more than three to six months [19]. Additionally, in this study, more than half of the SLP users used the product only in the evening (63.7%), a quarter (27.4%) of the SLP users used the product twice daily, morning and evening, only a minority used the product only in the morning. Compared to the South African study (40.1%) of the women reported using the product in the morning, and almost half (55.1%) used the product both morning and evening [19].

In a study conducted in Jordan, most users of SLPs get their products from pharmacies (52.6%) or from small cosmetics and skin care shops (31.8%) [20], which is similar to the results in our study where (72.6%) formerly bought these items from pharmacies. Given the well-known mercury, mercury products are readily available in pharmacies, online retailers, and beauty shops. Approximately 45% of the SLPs tested were found to have excessive levels of mercury well in excess of Food and Drug Administration (FDA) guidelines and have been marketed in the Saudi markets for over a decade [21]. This highlights the critical role pharmacists play in advising patients on skin-lightening treatments and providing them with instructions on how to use them.

Most of our participants (71.2%) agreed or strongly agreed that product price is an important criterion when choosing an SLP. A previous study in Saudi Arabia reported that the monthly cost of the bleaching products varied between 5 and 3000 Saudi Riyals (SR). Of the women, 16.7% reported spending more than SR200 per month on bleaching products ($53.4) [15].

In the current study, most SLP users (89.8%) believe that the effects of sun exposure counteract the effects of SLPs and (94.6%) that sunscreen should be applied when going outside, particularly while using SLPs. This awareness was higher than in previous studies in South Africa (69%) and the Middle East (63.5%) [19,20].

Regarding the participants' awareness of possible side effects caused by SLPs in the current study, most women (76%) believed that SLP could have adverse consequences and unwanted side effects on the skin. This result is similar to a study conducted in South Africa. Most respondents (89%) agreed that SLP could adversely affect the skin [22]. In contrast, a study conducted in northern Saudi Arabia reported that only 39.2% believed SLPs could harm their skin [6]. This discrepancy could be due to increasing awareness nowadays or the time frame of the study.

The use of SLP was associated with a very high level of satisfaction among respondents. In the current study, 75.4% of users are satisfied with the product's effectiveness. The rate found in our study is similar to that among Jordanian (70.9%), African, and Indian women [20,22]. In the other study conducted in Northern Saudi Arabia, respondents' satisfaction with the outcomes of their SLPs was 49.2% [6].

The role that lighter skin tone plays in mate choice, men's likely perception that lighter skin tones are more attractive, and the belief that lighter skin tone is seen as more beautiful are motivating factors responsible for using SLPs. Most respondents in various previous studies said they believed lighter skin was more attractive to men than darker skin and reduced their chances of marriage [6,20]. This roughly similar rate was found in our study, where more than half of respondents (58.7%) believe that lighter skin tone is perceived as more attractive by men and increases women's chances of getting married.

According to a recent study, the location of skin lesions, particularly in people with pigment disorders, is a key element affecting identity development, anxiety and depressive disorders, and depression [23]. Unlike populations in other nations [15,22], participants in this study tended to use creams on their faces (74.7%) and on non-sun-exposed areas (45%), consistent with another study conducted in Saudi Arabia, where participants used SLP on the face (36%) and non-sun-exposed areas (31%) [6].

Although hydroquinone is a commonly used skin-lightening agent, it is characterized by the side effects such as irritant dermatitis, contact dermatitis, melanocyte destruction, and ochronosis, which discolors the skin with repeated use over a long period [24]. Unfortunately, almost 80% of skin lightener users do not know if it is possible to use a hydroquinone bleaching product for over three months, making them more susceptible to these side effects.

According to our research, 44.4% of participants used the triple blend without a prescription. This result is similar to another survey when only 26% of the participants took it as recommended by the doctor [25]. There has been much discussion in most publications about the use of topical steroids without a prescription. For example, Jha et al. observed that 42.9% of patients bought topical corticosteroid (TC) creams without a prescription [26]. The literature has reported several side effects associated with TCs. They can be generally classified as local and systemic side effects. The immediate effects include stinging and irritation. In addition, the epidermis may be affected by atrophy, hypo/hyperpigmentation, photosensitivity, loss of skin barrier, and premature aging. Several factors can contribute to preventing these adverse effects of topical steroids, including prescribing, appropriate drug use, and proper counseling [27].

Study limitations

There are a few limitations in our study that should be taken into account when interpreting the results. First, some women may have provided misleading or false information in response to survey questions due to a variety of factors, including embarrassment over their honest reasons for using skin lighteners and possible knowledge of the illegality of some of the items used. Second, recall bias may have affected the results. Third, the study covered only one region in Saudi Arabia and, therefore, cannot be generalized to the whole country.

## Conclusions

This research shows that Saudi women frequently utilize topical lightening products. Many participants agreed that a lighter skin tone increases a woman's chances of getting married, and helps a woman get a better job opportunity which increases the consumption of those agents, and leads to several health issues. Therefore, regulation and controlling the use of bleaching products is important, as is educating women about the risks involved with this practice. The misuse of bleaching products should decline with greater awareness.
